# Quality of care for adult in-patients with malaria in a tertiary hospital in Uganda

**DOI:** 10.1186/s12936-021-03712-3

**Published:** 2021-04-09

**Authors:** Ronald Kiguba, Charles Karamagi, Sheila M. Bird

**Affiliations:** 1 Department of Pharmacology and Therapeutics, Makerere University College of Health Sciences, Kampala, Uganda; 2 Clinical Epidemiology Unit, Makerere University College of Health Sciences, Kampala, Uganda; 3 Medical Research Council Biostatistics Unit, Cambridge, UK

**Keywords:** Antimalarial use, Delayed initiation of antimalarials, Malaria diagnosis, Missed day 1 dosing, Risk factors, Incomplete dosing

## Abstract

**Background:**

Prompt detection and appropriate treatment of malaria prevents severe disease and death. The quality of care for adult malaria in-patients is not well documented in sub-Saharan Africa, particularly in Uganda. The study sought to describe the patterns of malaria diagnosis and treatment among adult in-patients admitted to the medical and gynaecological wards of Uganda’s 1790-bed Mulago National Referral Hospital from December 2013 to April 2014.

**Methods:**

A prospective cohort of 762 consented in-patients aged ≥ 18 years was assembled. Proportions of in-patients who received preadmission and in-hospital anti-malarials, missed Day 1 dosing of hospital-initiated anti-malarials and/or had malaria microscopy done were determined. Multivariable logistic regression was used to identify risk-factors for missed Day 1 dosing of anti-malarials.

**Results:**

One in five (19%, 146/762) in-patients had an admission or discharge malaria diagnosis or both; with median age of 29 years (IQR, 22–42 years). Microscopy was requested in 77% (108/141) of in-patients with an admission malaria diagnosis; results were available for 46% (50/108), of whom 42% (21/50) tested positive for *Plasmodium falciparum* malaria parasitaemia. Only 13% (11/83) of in-patients who received in-hospital injectable artesunate (AS) or quinine (Q) received follow-up oral artemether-lumefantrine (AL); 2 of 18 severe malaria cases received follow-up oral AL. Injectable AS only (47%, 47/100) was the most frequent hospital-initiated anti-malarial treatment followed by injectable Q only (23%, 23/100) amongst in-patients who received in-hospital anti-malarials. A quarter (25%, 25/100; 95% CI: 17–35%) of in-patients missed Day 1 dosing of hospital-initiated anti-malarials. Each additional admission diagnosis was more than two-fold likely to increase the odds of missed Day 1 dosing of in-hospital anti-malarials (aOR = 2.6, 95% CI: 1.52–4.56; *P*-value = 0.001).

**Conclusions:**

Half the malaria microscopy results were not available; yet, the rate of testing was high. The majority of in-patients initiated on injectable AS or Q did not receive the recommended follow-up oral AL. One in four in-patients delayed to initiate hospital anti-malarials by at least one calendar day. The hospital should encourage prompt availability of malaria test-results to promote the timely initiation and completion of anti-malarial treatment, thereby improving the quality of care for hospitalized malaria patients in Uganda.

**Supplementary Information:**

The online version contains supplementary material available at 10.1186/s12936-021-03712-3.

## Background

Around 409,000 people died from malaria globally in 2019, 94% of whom were from the World Health Organization (WHO) African Region. Prompt detection and appropriate treatment of malaria prevents severe disease and death [1]. The risk of mortality from severe malaria is highest during the first 24 h of hospitalization [2]. Yet, in most moderate- to high-malaria-transmission settings, long transit-time to a suitable health facility where appropriate intravenous anti-malarials can be administered could delay the initiation of appropriate anti-malarials and increase the risk of patient deterioration or death [2]. Other impediments to the timely initiation of appropriate anti-malarials include the lack of timely laboratory diagnosis and drug stock-outs [2, 3]. 

The WHO recommends confirmation of malaria diagnosis by quality microscopy or malaria rapid diagnostic testing within 2 h of patient presentation and before administration of anti-malarials. Otherwise, the decision to treat should be taken on clinical grounds. If severe malaria is suspected, parasitological diagnosis should not delay initiation of anti-malarials [2]. Adults with severe malaria, including pregnant women in all trimesters and breast-feeding mothers, should be treated with three doses of injectable artesunate (AS) for 24 h minimum at 0, 12 and 24 h regardless of whether the patient can tolerate oral treatment earlier. If unable to take oral medication, the patient should continue with injectable AS once daily, for a maximum of 7-days. If injectable AS is not available, once daily injectable artemether (AT) or 8-hourly injectable quinine (Q) should be administered. Following injectable anti-malarials, a full 3-day course of oral artemisinin-based combination therapy (ACT)—mainly artemether-lumefantrine (AL) (six doses) for Uganda—should be administered if the patient is able to take oral medication [2, 4–6]. Other recommended artemisinin-based combinations include artesunate-amodiaquine (AQ) and dihydroartemisinin-piperaquine (DP) (three doses for each combination). If full treatment for severe malaria is not possible at a given health facility but injectables are available, adults and children should be given one intramuscular dose of AS or Q and referred to a suitable facility for appropriate management [2].

It is necessary for patients with severe malaria to initiate timely appropriate anti-malarials and complete full courses of prescribed anti-malarials, which promotes therapeutic success, reduces malaria-related mortality and prevents drug resistance [7–9]. However, SSA patients with severe malaria frequently receive incomplete doses of prescribed anti-malarials and/or treatment meant for uncomplicated malaria [8, 10]. One-third of hospitalized Ugandan patients missed Day 1 of prescribed antibiotics [3], but similar data are scarce on anti-malarial use. This study aims to describe the patterns of malaria diagnosis and treatment [i.e. anti-malarial use by extent of use, missed opportunity for treatment, frequency of administered-treatment, medication-use-cycle (prescription-dispensing-administration), missed Day 1 dosing and mortality] among adult in-patients at Uganda’s 1790-bed Mulago National Referral Hospital. The study also evaluates patient-level risk-factors for missed Day 1 dosing of prescribed anti-malarials and the relationship between missed Day 1 dosing of prescribed anti-malarials and length of hospital stay among these Ugandan adult in-patients.

## Methods

Detailed account of the study design, setting, data collection and data management is documented elsewhere [3]. Briefly, key details are described below.

### Study design and setting

A prospective cohort study was conducted among in-patients, 18 years and older, at Mulago National Referral Hospital with bed capacity of 1790 and an annual in-patient turnover of 140,000 patients. Three medical wards and one Gynaecological ward were included, each with an official bed capacity of 54 and average of 5–25 patient admissions per day [3]. Not all women of childbearing age have a pregnancy test done.

Details on the prescription, dispensing and administration of medicines are hand-recorded in the patients’ charts. Hospital pharmacists dispense the prescribed free-of-charge injectable and/or oral anti-malarials, as appropriate, to in-patients/caregivers in quantities that discourage misuse of medicines. However, the in-patients/caregivers are instructed to return to the pharmacy early enough for more medication to avoid missing treatment [3].

### Data collection

The data were collected in December 2013 to April 2014 by four teams of trained research assistants. Each research team had a medical officer, clinical pharmacist and degree nurse. An internist on the medical wards and a gynaecologist/obstetrician on the gynaecology ward solved any clinical problems faced by the research assistants, while the principal investigator advised on pharmacological issues. The research team did not interrupt routine clinical care. Study patients provided written informed consent and were enrolled at the four wards using a systematic sampling procedure following a daily random start from the first two (Infectious Diseases and Gastrointestinal Illnesses ward), three (Haematology, Neurology and Endocrinology ward) and four (Cardiovascular, Pulmonology and Nephrology ward & Gynaecology ward) new admissions; and subsequently every second, third and fourth admission, respectively. Patients were assessed at baseline (demographics, clinical conditions, medications) and on a daily basis (clinical conditions, medications) until discharge, transfer, death, or loss to follow-up [3].

Research assistants captured clinical data from patients’ clinical charts and interviewed the patients, their caregivers and/or ward staff. Each research team's medical officer clerked the in-patients to obtain additional clinical data not documented in the clinical charts. Each research team’s pharmacist captured baseline medication data at the time of hospital admission by interviewing the in-patients and reviewing all available medical documents. During hospital stay, research pharmacists obtained medication data from the patients’ clinical charts, ward pharmacy records, pill counts of patients’ oral medications (tablets, capsules), views of unused parenteral medicines possessed by the patients/caretakers and daily interviews with the patients/caregivers and/or ward staff. The data were collected daily from 8.00am to 6.00 pm from Monday to Friday and from10.00am to 6.00 pm on weekends and public holidays [3].

### Data management

Epidata 3.1 software was programmed with checks to limit data entry errors and the electronic database password-secured to limit access to authorized personnel only [3].

### Statistical analysis

#### Patterns of malaria diagnosis and anti-malarial use

The proportions of in-patients who received anti-malarials preadmission and during hospitalization were determined using, as numerator, the number of in-patients who received at least one anti-malarial and, as denominator, the total number of study in-patients. We calculated the proportions of in-patients with available (positive, negative) and unavailable (not returned, not requested) malaria microscopy results and those who experienced missed Day 1 dosing of hospital-initiated anti-malarials. See Additional file 1 for details of the analysis plan for post-admission time-to-first-dose among in-patients with an admission malaria diagnosis; and parenteral-to-oral-switch of anti-malarials.

Chi-squared tests were used to screen univariate-level relationships between patient-level characteristics and anti-malarial use during hospitalization (yes/no); and potential patient-level risk-factors for missed Day 1 anti-malarial dosing during hospitalization. Multivariable logistic regression was used to identify risk-factors for missed Day 1 dosing of anti-malarials. Results were expressed as odds ratios (ORs) with their 95% confidence intervals (CIs). Poisson CIs were used for counts below 16. Stata 14.0 was used for all the analyses [11].

### Identification of missed Day 1 dosing of anti-malarials

Among in-patients for whom an anti-malarial was prescribed during the current hospitalization and at least one dose was administered, missed Day 1 dosing was measured in two ways; (i) calendar-day as proposed by Kiguba et al. [3] and (ii) 24-h timescale using date-and-time of hospital admission and date-and-time of first in-hospital anti-malarial dose.

## Results

### Study population

**Fig. 1:**
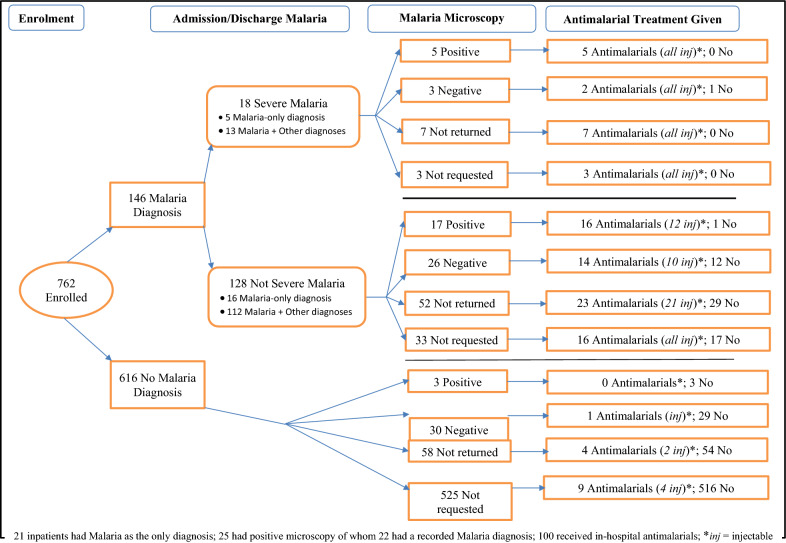
Schema of enrolment, malaria diagnosis and antimalarial treatment among 762 in-patients at a tertiary care hospital, Uganda

*Demographic and clinical characteristics:* The median age of 762 in-patients was 30 years (interquartile range, IQR, 24–42 years), see Table 1. About one in five (19%, 141/762; 95% CI: 16–21%) in-patients had an admission malaria diagnosis, see Tables 1 & 2. About one in eight (12%, 88/762; 95% CI: 9–14%) in-patients had a discharge malaria diagnosis: 44% (39/88; 95% CI: 34–55%) had malaria as their single discharge diagnosis; recorded as severe malaria in 10 of 39 in-patients. About one-fifth (19%, 146/762; 95% CI: 16–22%) of in-patients had an admission or discharge malaria diagnosis or both, and median age of 29 years (IQR, 22–42 years); of whom 15% (21/146; 95% CI: 9–21%) had a single admission/discharge malaria diagnosis, see Tables 2 & Additional file 1: Table S1 and Fig. 1, and 21% (30/146; 95% CI: 14– 28%) had malaria-in-pregnancy. Eleven of the 21 in-patients with a single admission/discharge malaria diagnosis had malaria-in-pregnancy, see Additional file 1: Table S1; 20 of the 21 were female. Severe malaria was recorded for 12% (18/146, 95% CI: 7–19%) of in-patients with an admission/discharge malaria diagnosis, see Fig. 1.

**Table 1:** Demographic and clinical characteristics of 762 in-patients, Uganda

Characteristic	Anti-malarial Use during the Current Hospitalization		
	Yes	No	Overall
Age, years^a^	27 (21–35)	30 (25–43)	30 (24–42)
Length of hospital stay, days^a^	4 (3–5)	4 (3–6)	4 (3–6)
Patient-days of observation	454	3287	3741

Extent of anti-malarial use	Anti-malarial Use during the Current Hospitalization, n (%)		
	Yes	No	Total
Pre-admission anti-malarials	97 (13)	665 (87)	762
In-hospital anti-malarials	100 (13)	662 (87)	762
Pre-admission anti-malarials	38 (38)	62 (62)	100
Pre-/in-hospital co-trimoxazole	15 (15)	85 (85)	100
In-hospital antibiotics	61 (61)	39 (39)	100
In-hospital antiretrovirals	14 (14)	86 (86)	100
Pre-/in-hospital anti-malarials	159 (21)	603 (79)	762

Subgroup analyses on key variables	Anti-malarial Use, n (%)			Single factor analysis		
	Yes	No	Total, [% col]^b^	OR^c^	95% CI^d^ for OR	*P-*value
Gender						
Male	20 ( 9)	208 (91)	228 [30]	1.0		
Female	80 (15)	454 (85)	534 [70]	1.8	1.09–3.07	0.022
Ward						
Gynaecological (GYN)	25 (13)	166 (87)	191 [25]	1.0		
Infectious Diseases and Gastrointestinal Illnesses (IDGI)	57 (18)	263 (82)	320 [42]	1.4	0.87–2.39	0.161
Haematology, Neurology and Endocrinology (HNE)	12 (10)	105 (90)	117 [15]	0.8	0.37–1.58	0.459
Cardiovascular, Pulmonology and Nephrology (CPN)	6 ( 4)	128 (96)	134 [18]	0.3	0.12–0.78	0.013
Number of working diagnoses						
One	21 (15)	122 (85)	143 [18]	1.0		
Two	26 (13)	177 (87)	203 [27]	0.9	0.46–1.59	0.616
Three	28 (15)	158 (85)	186 [24]	1.0	0.56–1.90	0.926
Four or more	25 (11)	205 (89)	230 [30]	0.7	0.38–1.32	0.277
Length of hospital stay, days						
Less than 5-days	64 (15)	368 (85)	432 [57]	1.0		
Five days or more	36 (11)	294 (89)	330 [43]	0.7	0.46–1.09	0.115
HIV-serostatus						
Negative	49 (14)	291 (86)	340 [45]	1.0		
Positive	23 (10)	209 (90)	232 [30]	0.7	0.39–1.11	0.113
Unknown	28 (15)	162 (85)	190 [25]	1.0	0.62–1.70	0.919
Hospitalization in past 3-months						
No	75 (14)	455 (86)	532 [70]	1.0		
Yes	25 (11)	205 (89)	230 [30]	0.7	0.46–1.20	0.227
Charlson's co-morbidity index score						
Zero	64 (16)	329 (84)	393 [52]	1.0		
One or more	36 (10)	333 (90)	369 [48]	0.6	0.36–0.86	0.008
Antiretroviral therapy use						
No	86 (14)	549 (86)	635 [83]	1.0		
Yes	14 (11)	113 (89)	127 [17]	0.8	0.43–1.44	0.444
Microscopy—Malaria Parasitaemia Results Available						
No	62 (62)	616 (93)	678 [89]	1.0		
Yes	38 (38)	46 ( 7)	84 [11]	8.2	4.81–14.0	< 0.001
Major admission diagnosis						
Malaria						
No	17 ( 3)	604 (97)	621 [81]	1.0		
Yes	83 (59)	58 (41)	141 [19]	50	28.3–91.5	< 0.001
Immunosuppressed syndrome (ISS) or HIV/AIDS^e^						
No	86 (14)	524 (86)	610 [80]	1.0		
Yes	14 ( 9)	14 (91)	152 [20]	0.6	0.34–1.12	0.113
Tuberculosis (TB)						
No	92 (14)	548 (86)	640 [84]	1.0		
Yes	8 ( 7)	114 (93)	122 [16]	0.4	0.20–0.88	0.023
Sepsis-related working diagnosis						
No	81 (12)	597 (88)	678 [89]	1.0		
Yes	19 (23)	65 (77)	84 [11]	2.2	1.23–3.78	0.007
Respiratory Conditions except TB						
No	85 (13)	547 (87)	632 [83]	1.0		
Yes	15 (12)	115 (88)	130 [17]	0.8	0.47–1.51	0.557
Miscellaneous infections						
No	78 (12)	571 (88)	649 [85]	1.0		
Yes	22 (19)	91 (81)	113 [15]	1.8	1.05–2.98	0.032

^a^Median (Interquartile Range, IQR)

^b^% Column

^c^*OR* Odds Ratio

^d^confidence interval

^e^Not all HIV-positive patients had the immunosuppressed syndrome, ISS

**Table 2:** Malaria detection by laboratory diagnosis among 762 hospitalized patients, Uganda ^±^

Malaria suspected at admission (n = 141) ^~^																Malaria not suspected at admission (n = 621)															
Malaria at discharge (n = 83)								No malaria at discharge (n = 58)								Malaria at discharge (n = 5)								No malaria at discharge (n = 616)							
Microscopy requested, n (%) ^¥^																															
Yes						No		Yes						No		Yes						No		Yes						No	
Returned Positive		Returned Negative		Not Returned		Not Requested		Returned Positive		Returned Negative		Not Returned		Not Requested		Returned Positive		Returned Negative		Not Returned		Not Requested		Returned Positive		Returned Negative		Not Returned		Not Requested	
21 (25)		13 (16)		31 (37)		18 (22)		0 (0)		16 (28)		27 (47)		15 (26)		1 (20)		0 (0)		1 (20)		3 (60)		3 (0)		30 (5)		58 (9)		525 (85)	
In-hospital administration of anti-malarials, n (%)																															
Yes	No	Yes	No	Yes	No	Yes	No	Yes	No	Yes	No	Yes	No	Yes	No	Yes	No	Yes	No	Yes	No	Yes	No	Yes	No	Yes	No	Yes	No	Yes	No
20 (95)	1 (5)	11 (85)	2 (15)	26 (84)	5 (16)	15 (83)	3 (17)	0 (0)	0 (0)	5 (31)	11 (69)	3 (11)	24 (89)	3 (20)	12 (80)	1 (100)	0 (0)	0 (0)	0 (0)	1 (100)	0 (0)	1 (33)	2 (67)	0 (0)	3 (100)	1 (3)	29 (97)	4 (7)	54 (93)	9 (2)	516 (98)
Single admission diagnosis of malaria, n (%)																															
Yes	No	Yes	No	Yes	No	Yes	No	Yes	No	Yes	No	Yes	No	Yes	No	Yes	No	Yes	No	Yes	No	Yes	No	Yes	No	Yes	No	Yes	No	Yes	No
5 (24)	16 (76)	0 (0)	13 (100)	5 (16)	26 (84)	4 (22)	14 (78)	0 (0)	0 (0)	1 (6)	15 (94)	1 (4)	26 (96)	0 (0)	15 (100)	0 (0)	1 (100)	0 (0)	0 (0)	0 (0)	1 (100)	0 (0)	3 (100)	0 (0)	3 (100)	0 (0)	30 (100)	0 (0)	58 (100)	0 (0)	525 (100)
Received anti-malarials during the 4-weeks preadmission, n (%)																															
Yes	No	Yes	No	Yes	No	Yes	No	Yes	No	Yes	No	Yes	No	Yes	No	Yes	No	Yes	No	Yes	No	Yes	No	Yes	No	Yes	No	Yes	No	Yes	No
7 (33)	14 (67)	5 (38)	8 (62)	8 (26)	23 (74)	7 (39)	11 (61)	0 (0)	0 (0)	5 (31)	11 (69)	8 (30)	19 (70)	3 (20)	12 (80)	0 (0)	1 (100)	0 (0)	0 (0)	1 (100)	0 (0)	1 (33)	2 (67)	0 (0)	3 (100)	10 (33)	20 (67)	5 (9)	53 (91)	37 (7)	488 (93)
Single discharge diagnosis of malaria, n (%)																															
Yes	No	Yes	No	Yes	No	Yes	No	Yes	No	Yes	No	Yes	No	Yes	No	Yes	No	Yes	No	Yes	No	Yes	No	Yes	No	Yes	No	Yes	No	Yes	No
9 (43)	12 (57)	3 (23)	10 (77)	16 (52)	15 (48)	11 (61)	7 (39)	0 (0)	0 (0)	0 (0)	16 (100)	0 (0)	27 (100)	0 (100)	15 (0)	0 (0)	1 (100)	0 (0)	0 (0)	0 (0)	1 (100)	0 (0)	3 (100)	0 (0)	3 (100)	0 (0)	30 (100)	0 (0)	58 (100)	0 (0)	525 (100)

^**±**^19% (146/762; 95% confidence interval (CI): 16–22%) of in-patients had either an admission or discharge malaria diagnosis or both; ^**~**^19% (141/762; 95% CI: 16–21%) had an admission malaria diagnosis; ^¥^Only three of the 201 in-patients with microscopy requests had concurrent malaria rapid diagnostic testing (mRDT) done. However, mRDT results were available for only one in-patient, who tested positive

### Laboratory diagnosis of malaria

Microscopy was requested in 26% (201/762) of in-patients; laboratory results were available for 42% (84/201; 95% CI: 34–48%) of them, of whom 30% (25/84; 95% CI: 20–41%) tested positive, see Fig. 1. Microscopy was requested in 77% (108/141; 95% CI: 69–83%) of in-patients with an admission malaria diagnosis; laboratory results were available for 46% (50/108; 95% CI: 37–56%) of them, of whom 42% (21/50; 95% CI: 28–57%) tested positive, see Table 2 and Additional file 1. At bivariate level, in-patients with an admission malaria-in-pregnancy diagnosis were ten-fold more likely to test positive for malaria when compared with non-pregnancy-related malaria in-patients (odds ratio, OR 10.1; 95% CI: 1.55–65.96; 1 degree of freedom, df; χ2 = 9; *P*-value = 0.003) i.e. [82% (9/11; 95% CI: 48–98%) vs. 31% (12/39; 95% CI: 17–48%)], respectively.

### Extent of anti-malarial use

Thirteen percent (97/762; 95% CI: 10–15%) of in-patients received anti-malarials during the 4-weeks pre-admission, see Table 1 of whom 44% (43/97; 95% CI: 34–55%) had an admission malaria diagnosis. Thirteen percent (100/762; 95% CI: 11–16%) of in-patients received anti-malarials during the current hospitalization, see Table 1 of whom 83% (83/100; 95% CI: 74–90%) had an admission malaria diagnosis, see Table 1.

### Missed opportunity for hospital-initiated anti-malarials

Four of 25 (16%, 95% CI: 5–36%) in-patients with a positive malaria test did not receive in-hospital anti-malarials, see Fig. 1. No admission/discharge malaria diagnosis was recorded for three of the four in-patients, see Fig. 1, two of whom had a malignancy; the fourth in-patient had poorly treated malaria on admission and run away from hospital due to delayed investigations and treatment, see Table S1. None of the in-patients died while in hospital, see Box 1; none had malaria-in-pregnancy.

**Box 1:** Missed opportunity for hospital-initiated anti-malarial treatment for four in-patients with malaria parasitaemia as confirmed by microscopy, Uganda

Particulars	Clinical notes
Patient 1	A 60-year-old female with unknown HIV-status, 6-year history of hypertension and type 2 diabetes mellitus (DM) presented with poorly controlled DM having defaulted on DM treatment for 8-months. Microscopy for malaria parasites was requested on the day of admission (Day 1). Results were returned on Day 1 with confirmed malaria parasitaemia. AL and paracetamol were prescribed on Day 2 but not dispensed. The patient was discharged on Day 3 without anti-malarial treatment
Patient 2	A 24-year-old female with unknown HIV-status was referred from a clinic where she had been treated for suspected malaria and typhoid with no improvement. She presented with poorly treated malaria and microscopy for malaria parasites was requested on Day 1. Results were returned on Day 2 with confirmed malaria parasitaemia. AL and paracetamol were prescribed on Day 2 but not dispensed and the patient was discharged on Day 2 without anti-malarial treatment
Patient 3	A 44-year-old HIV-negative male was transferred from a referral hospital. He presented with an admission diagnosis of chronic lymphocytic leukaemia and confirmed malaria parasitaemia by microscopy. No fresh request for malaria microscopy was made during the current admission. The patient did not receive any anti-malarial treatment prescription and/or administration both prior to admission and throughout the current hospitalization. He was transferred to Uganda Cancer Institute on Day 3
Patient 4	A 43-year-old HIV-positive female with history of DM who was receiving second-line antiretroviral therapy (tenofovir, lamivudine, lopinavir/ritonavir) and co-trimoxazole presented with an admission diagnosis of colon cancer. Microscopy for malaria parasites was requested on Day 2 and results were returned the same day with confirmed malaria parasitaemia. No anti-malarial treatment was prescribed, dispensed or administered during hospitalization. The patient continued to receive her antiretrovirals and co-trimoxazole; and was transferred to Uganda Cancer Institute on Day 17

### Frequency of administered anti-malarials

*Four-week preadmission period*: At patient-level, oral artemether-lumefantrine (AL) only (52%, 50/97; 95% CI: 41–62%) was most frequently administered followed by injectable quinine (Q) only (23%, 22/97; 95% CI: 15–32%), see Table 3 and Appendix.

**Table 3:** Frequency of anti-malarials used by hospitalized patients, Uganda, 2014

Anti-malarial	Number	n, %
Patient-level		
Pre-admission, n = 97		
Artemether-Lumefantrine only	50	52
Quinine only	22	23
Sulfadoxine-Pyrimethamine only	9	9
Artesunate only	5	5
Coartem + Quinine only	4	4
Duocotecxin only	2	2
Artemether only	1	1
Artesunate + Duocotexcin only	1	1
P-alaxin + Quinine only	1	1
Artemether + Quinine + Doxycycline only	1	1
Dihydroartemisinin-Piperaquine only	1	1
In-hospital, n = 100*		
Artesunate only^±^	47	47
Quinine only^~^	23	23
Artemether-Lumefantrine only	15	15
Artesunate + Artemether-Lumefantrine only		
Quinine + Artemether/Lumefantrine only	3	3
Sulfadoxine-Pyrimethamine only	2	2
Artesunate + Quinine only	2	2
Drug-level		
Pre-admission, n = 105		
Artemether-Lumefantrine	54	51
Quinine	28	27
Sulfadoxine-Pyrimethamine	9	9
Artesunate	6	6
Duocotexcin	3	3
Artemether	2	2
Dihydroartemisinin-Piperaquine	2	2
Doxycycline	1	1
In-hospital, n = 113		
Artesunate	57	50
Quinine	28	25
Artemether-Lumefantrine	26	23
Sulfadoxine-Pyrimethamine	2	2

^*^Only 13% (11/83) of in-patients who received injectable artesunate or quinine received follow-up oral artemether-lumefantrine; ^±^68% (32/47) of the in-patients presented with both admission and discharge malaria diagnoses [median length of hospital stay: 4 (IQR, 3 to 5) days]; ^~^91% (21/23) of the in-patients had both admission and discharge malaria diagnoses [median length of hospital stay: 3 (IQR, 2 to 4) days]

*Current hospitalization*: At patient-level, injectable AS only (47%, 47/100; 95% CI: 37–57%) was the most frequently administered followed by injectable Q only (23%, 23/100; 95% CI: 15–32%), oral AL only (15%, 15/100; 95% CI: 9–24%) and AS + AL only (8%, 8/100; 95% CI: 4–15%), among others; see Table 3 and Additional file 1.

### Medication-use-cycle

#### Overview of prescription, dispensing and administration of anti-malarials

*Overall:* Anti-malarials were prescribed for 15% (114/762) of in-patients, dispensed to 79% (90/114), yet, administered in 100 in-patients (93 of 100 had an anti-malarial prescription), see Additional file 1 for details on AS, Q and AL.

### Incomplete dosing of in-hospital anti-malarials

*Artesunate:* 25% (14/57; 95% CI: 14–38%) of in-patients in whom in-hospital AS was administered received < 3 doses of both dispensed and administered AS irrespective of pregnancy, see Additional file 1.

*Quinine:* 21% (6/28; 95% CI: 8–41%) of in-patients in whom in-hospital Q was administered received < 3 doses of both dispensed and administered Q irrespective of pregnancy, see Appendix.

*Artemether-Lumefantrine:* 71% (20/28; 95% CI: 51–87%) of in-patients in whom *in-hospital AL was administered* received < 6 doses of *administered AL*.

*Artesunate or Quinine* + *Artemether-Lumefantrine:* About 13% (11/83) of the in-patients who received in-hospital injectable AS or Q also received at least one dose of follow-up oral AL during the current hospitalization, see Table 3; AL having been co-prescribed for 48 (58%) of the 83 in-patients. AL was co-prescribed for 12 (67%) of the 18 severe malaria cases and administered in 2 cases only during the current hospitalization.

### Missed Day 1 dosing of hospital-prescribed anti-malarials

*Overall:* A quarter (25%, 25/100; 95% CI: 17–35%) of in-patients who received anti-malarials during the current hospitalization missed Day 1 dosing of hospital-initiated anti-malarials based on calendar-day. A similar estimate of missed Day 1 dosing was obtained based on post-admission 24-h-delay, see Appendix.

*Artesunate:* Around a quarter (28%, 16/57; 95% CI: 17–42%) of in-patients who initiated anti-malarials with AS missed Day 1 dosing based on calendar-day, see Table 4.

**Table 4:** Missed Day 1 dosing of artesunate injection among 57 hospitalized patients who received in-hospital intravenous artesunate, Uganda, 2014

Length of stay, days	Preadmission anti-malarial use	Admission Malaria Diagnosis	Discharge Malaria Diagnosis	AL co-prescribed	Switched to AL	AS Doses Prescribed	AS Doses Received	Day 1	Day 2	Day 3	Day 4	Day 5	Day 6	Day 7	Day 8
3	No	Yes	No	No	No	3	1		1 dose						
3	No	Yes	Yes	Yes	No	2	3	1 dose	2 doses						
4	No	Yes	Yes	No	No	6	2	1 dose	1 dose						
5	No	Yes	Yes	Yes	No	3	3	1 dose	1 dose	1 dose					
2	No	Yes	Yes	Yes	No	2	1	1 dose							
4	Yes	Yes	Yes	Yes	No	10	3	1 dose		1 dose	1 dose				
5	No	No	No	No	No	3	2		2 doses						
2	No	Yes	Yes	Yes	Yes	3	2	2 doses							
4	No	Yes	No	Yes	Yes	3	2	1 dose	1 dose						
5	Yes	Yes	Yes	Yes	Yes	3	1	1 dose							
4	No	Yes	Yes	Yes	No	3	2	1 dose	1 dose						
5	No	Yes	Yes	No	No	3	4		1 dose	2 doses	1 dose				
5	No	Yes	Yes	No	No	3	3	1 dose	1 dose	1 dose					
5	No	Yes	No	No	No	3	3		1 dose	1 dose	1 dose				
10	Yes	Yes	No	No	No	3	2	1 dose	1 dose						
3	Yes	No	No	No	No	2	3	1 dose	2 doses						
5	Yes	Yes	Yes	Yes	No	2	2	1 dose	1 dose						
2	No	Yes	Yes	Yes	No	3	2	1 dose	1 dose						
3	No	Yes	Yes	Yes	No	2	3	2 doses	1 dose						
10	No	No	No	No	No	3	4		1 dose	1 dose	2 doses				
3	No	Yes	Yes	Yes	No	3	2		2 doses						
5	No	No	Yes	Yes	No	2	3		1 dose	1 dose	1 dose				
2	No	Yes	Yes	Yes	No	4	1	1 dose							
3	Yes	Yes	Yes	Yes	No	4	3	1 dose	1 dose	1 dose					
3	No	Yes	Yes	Yes	No	3	3	1 dose	2 doses						
3	Yes	Yes	Yes	No	No	3	3	1 dose	1 dose	1 dose					
3	Yes	Yes	Yes	Yes	No	3	2	1 dose	1 dose						
5	Yes	Yes	Yes	Yes	No	3	4	1 dose	1 dose	1 dose	1 dose				
4	Yes	Yes	Yes	Yes	No	3	3	1 dose	1 dose	1 dose					
3	Yes	Yes	Yes	Yes	No	3	3	1 dose	1 dose	1 dose					
5	Yes	Yes	Yes	Yes	No	3	3	1 dose		1 dose	1 dose				
7	Yes	Yes	Yes	Yes	Yes	3	3	1 dose				1 dose	1 dose		
5	No	No	No	Yes	No	3	3		1 dose	1 dose	1 dose				
3	Yes	Yes	Yes	Yes	No	3	2	1 dose	1 dose						
4	No	Yes	Yes	No	No	3	2	1 dose	1 dose						
2	No	Yes	Yes	Yes	No	3	1	1 dose							
9	No	Yes	Yes	No	No	3	2	1 dose	1 dose						
6	No	Yes	Yes	No	No	3	3				1 dose	2 doses			
3	Yes	No	No	Yes	No	3	1	1 dose							
4	No	Yes	Yes	Yes	No	3	3		1 dose	2 doses					
3	Yes	Yes	No	Yes	No	3	3	1 dose	2 doses						
4	No	Yes	Yes	No	No	3	3	1 dose	1 dose	1 dose					
3	Yes	No	Yes	Yes	No	3	3	1 dose	2 doses						
4	Yes	Yes	Yes	Yes	No	3	3		1 dose	2 doses					
9	No	Yes	Yes	Yes	Yes	4	3			1 dose	1 dose	1 dose			
4	Yes	Yes	No	Yes	Yes	2	3		2 doses	1 dose					
4	No	Yes	Yes	Yes	No	6	2			1 dose	1 dose				
3	No	Yes	Yes	Yes	Yes	1	1	1 dose							
16	Yes	Yes	No	No	No	3	3	1 dose	2 doses						
4	No	Yes	Yes	No	No	3	1	1 dose							
6	No	Yes	No	No	No	3	4	1 dose	1 dose	2 doses					
5	No	Yes	Yes	Yes	No	3	3		2 doses	1 dose					
3	No	Yes	No	No	No	1	1	1 dose							
3	No	Yes	Yes	Yes	No	3	1		1 dose						
4	Yes	No	No	No	No	2	2	1 dose	1 dose						
15	Yes	No	Yes	No	No	3	8	1 dose	2 doses		1 dose	2 doses			2 doses
1	No	Yes	Yes	Yes	No	2	2	1 dose	1 dose						
Missed-dose day, n								16	10	8	1	1			
Dose-day data unavailable, n1								0	5	27	44	52			
Dose-day data available, N*								57	52	30	13	5			
Proportion of in-patients with missed Day 1 data, n/N ~								28%							

The variation from 1 to 2 AS doses per calendar day depends on the time of day that an in-patient initiates treatment. Injectable AS is given at 0, 12, 24 h then in-patient is switched to oral AL if he/she can tolerate it. Thus, an in-patient might receive 1 to 2 AS doses per calendar-day and very rarely 3 AS doses

*N = [(The 57 artesunate users) – (Number of in-patients who did not have artesunate dose-day data)] or (57-n1); ~ 95% confidence intervals for the estimate is 28% (17–42%)

*Quinine:* About one in five (18%, 5/28; 95% CI: 6–37%) of in-patients who initiated anti-malarials with Q missed Day 1 dosing based on calendar-day, see Additional file 1: Table S2.

### Mortality among in-patients who received in-hospital anti-malarials

Four of 100 in-patients who received in-hospital anti-malarials died during hospitalization. All four in-patients had clinically-diagnosed malaria: microscopy was requested is three in-patients, but results were not available, see Box 2. An unconscious 88-year-old female of unknown HIV-status presented with a single admission diagnosis of severe malaria and pulse rate of 98 beats per minute. She received a pre-referral intramuscular Q dose 23 h preadmission and two Q doses 11 h apart after admission. She died on Day 2 of hospitalization. The other three cases had multiple diagnoses, see Box 2.

**Box 2:** Mortality of four in-patients who received in-hospital anti-malarial treatment, Uganda

Particulars	Clinical notes
Quinine	One in-patient who received Q during admission died in hospital
Patient 1-Q	An 88-year-old female of unknown HIV-status presented with a single admission diagnosis of severe malaria which manifested with fever, chills and unconsciousness. She was referred from a clinic for further management after receiving an initial intramuscular dose of quinine (23 h prior to the current admission). Her vitals on admission were: pulse rate (98 beats per minute); blood pressure (116/63 mmHg); temperature (35.9 °C). Microscopy for malaria parasites was requested on admission (Day 1) but the results were not returned by Day 2. She received 2-doses of Q which were administered 11 h apart, the first dose being 2 h after admission on Day 1. The patient died on Day 2 of hospitalization
Artesunate	Two in-patients who received AS during admission died in hospital. None of the two in-patients presented with either an admission or a definitive malaria diagnosis:
Patient 1-AS	A 20-year-old female of unknown HIV-status was admitted with suspected severe sepsis of chest focus, bacterial pneumonia, urinary tract infection (UTI), salmonellosis and acute gastroenteritis. Microscopy for malaria parasites was requested on Day 1 but the results were not returned. She missed Day 1 dosing of AS and subsequently received 4 doses of AS. Her definitive diagnoses were UTI, pneumonia and salmonellosis. She died on Day 4 of hospitalization
Patient 2-AS	A 24-year-old HIV-positive female presented with severe immunosuppression, sepsis, disseminated tuberculosis and/or tuberculous meningitis, atypical measles syndrome and toxoplasmosis. Microscopy for malaria parasites was not requested on admission. She received 2 doses of AS and never missed Day 1 dosing. Her definitive diagnosis was severe immunosuppression. She died on Day 10 of hospitalization
Artemether + Lumefantrine	One in-patient who received AL in hospital died
Patient 1-AL	A 23-year-old HIV-positive female presented with working diagnoses of immunosuppression, malaria, septicaemia, urinary tract infection and anaemia. Microscopy for malaria parasites was requested on admission (Day 1) but the results were not returned. Duocotexcin (DP) was prescribed on Day 1 but was neither dispensed nor administered. One dose of AL was administered on Day 3. Her definitive diagnoses were immunosuppression and malaria. She died on Day 6 of hospitalization

### Patient-level risk-factors for missed Day 1 dosing of anti-malarials

Number of admission diagnoses was a statistically significant risk-factor for missed Day 1 dosing of hospital-initiated anti-malarials based on calendar-day (aOR = 2.6, 95% CI: 1.52–4.56; *P*-value = 0.001), see Table 5. Similar results of missed Day 1 risk-factor were obtained based on post-admission 24-h-delay, see Table S3. Malaria-in-pregnancy and severity of malaria were not significantly related to missed Day 1 dosing of anti-malarials.

**Table 5:** Patient-level risk-factors for missed Day 1 dosing of administered anti-malarials based on calendar-day delay among 83 in-patients with an admission malaria diagnosis, Uganda, 2014

Missed Day 1 dosing of anti-malarials by calendar-day, n (%); (N = 83)												
Factor	Missed Calendar-Day 1 dosing				Crude Analysis				Adjusted Analysis			
	Yes	No	Total, [% col]^a^	OR^b^	95% for CI^c^	*P*-value		aOR^b^		95% for CI^c^		*P*-value
Antiretroviral therapy use												
No	15 (20)	59 (80)	74 [89]	1.0				1.0				
Yes	5 (56)	4 (44)	9 [11]	4.9	1.17–20.6		0.029	4.9		0.90–26.1	0.066	
Malaria microscopy test results available												
No	8 (17)	39 (83)	47 [57]	1.0				1.0				
Yes	12 (33)	24 (67)	36 [43]	2.4	0.87–6.82		0.090	2.4		0.70–8.40	0.165	
Linear on number of working												
Diagnoses	20	63	83	2.6	1.56–4.35		< 0.001	2.6		1.52–4.56	0.001	

^a^% Column

^b^*OR* Odds Ratio

^c^Confidence interval

## Missed Day 1 dosing of hospital-initiated anti-malarials versus length of hospital stay

No statistically significant association was observed between missed Day 1 dosing of anti-malarials and length of hospital stay (OR 1.1, 95% CI: 0.91–1.27; *P*-value < 0.396). The mean length of hospital stay for missed Day 1 cases was 4.7 (SD = 1.7) days *versus* 4.2 (SD = 2.5) days for non-cases.

## Discussion

Malaria microscopy was requested in 77% of in-patients with an admission malaria diagnosis, similar to estimates for the public sector (80%) in moderate- to high-transmission countries in sub-Saharan Africa (SSA) [1]. Unfortunately, only half the microscopy results were available to guide appropriate anti-malarial treatment. Thus, despite decent microscopy rates, healthcare professionals still rely on clinical judgement to treat half the suspected malaria cases. Clinical judgement increases the risk of unnecessary anti-malarial treatment and, in turn, depletes anti-malarial stocks for in-patients who truly need them; and increases the incidence of associated adverse drug reactions and drug resistance [2]. Seven in ten in-patients with suspected non-pregnancy-related malaria tested negative for malaria and would therefore not need anti-malarials; compared with only two in ten in-patients with suspected malaria-in-pregnancy. The value of a confirmed malaria diagnosis depends on prompt availability of parasitology results and whether the clinician uses the results to decide how to manage the patient. Malaria negative test-results as confirmed by microscopy—the gold standard—should prompt clinicians to examine patients for other causes of illness and treat them accordingly [2]. However, the interpretation of negative microscopy results should take into account the high rates of anti-malarial pre-treatment, which was as high as one in three admitted patients with suspected malaria in this patient cohort. A rapid diagnostic test (RDT), in addition to microscopy, could be used to detect the *HRP2* malaria antigen in patients who recently received anti-malarials and whose blood films are, thus, likely to show no malaria parasitaemia [2]. RDTs can give positive results for up to 1-month after parasite clearance [2].

One in six cases of confirmed malaria, none of whom had severe symptoms, did not receive anti-malarials during the current hospitalization, which raises concern over the safety of in-patient care at this tertiary care hospital. In high transmission zones, many patients with other causes of admission could carry malaria parasites without symptoms; however, they should receive anti-malarial treatment when the infection is confirmed. Poor coordination between the laboratory and clinicians is likely to lead to missed anti-malarial treatment, which is exacerbated by high in-patient loads of up to 80 admissions in wards with official bed capacity of 54 [3]. Introducing an integrated electronic health record (EHR) system to track in-patient care could significantly improve the flow of information between different hospital departments and, in so doing, promote efficient clinical management of in-patients [12].

Seven in eight in-patients initiated on injectable AS or Q did not receive the recommended follow-up oral AL. Also, one in four in-patients who received at least one in-hospital dose of prescribed anti-malarials missed the first day of their anti-malarial treatment. The missed treatment could be worsened by the observed disparities in prescribed, dispensed and administered anti-malarials—similar to observations made elsewhere [8, 10]. Possible reasons for these system lapses include; (i) drug stock-outs, (ii) poor communication between clinician and patient/caregiver and, (iii) work overload [3]. The hospital should improve its stock forecasting for in-demand anti-malarials, promote intern-pharmacist-led bedside dispensing to reduce the clinicians’ workload during drug administration, and improve supervision of junior and mid-level clinicians to promote accountability to in-patients and the hospital [3].

Each additional admission diagnosis increases by more than two-fold the odds of missed Day 1 dosing of prescribed anti-malarials, which underlines the need for prompt availability of malaria test-results to promote the timely initiation of anti-malarials. Prompt and complete anti-malarial treatment rapidly eliminates malaria parasites from a patient’s bloodstream [13]. Patients with severe malaria should access timely appropriate anti-malarials and complete full courses of prescribed anti-malarials to promote therapeutic success, reduce malaria-related morbidity and mortality, and prevent the emergence and spread of drug resistance [7–9].

In-patients with an admission malaria-in-pregnancy diagnosis seemed more likely to have a microscopically-confirmed malaria diagnosis than in-patients with other admission malaria diagnoses. This comparative advantage at diagnosis did not translate into better anti-malarial treatment because no pregnancy-related difference was observed in the prescription, dispensing and administration of anti-malarials. Improvement in the anti-malarial medication-use-cycle should target systemic weaknesses.

Unlike Q, the hospital frequently encounters drug stock-outs of in-demand free-of-charge AS and AL, which in-patients must purchase from private community pharmacies to prevent lapses in prescribed treatment. Drugs bought from private community pharmacies are not recorded as dispensed in the hospital pharmacy registers [3], which explains why the reported number of in-patients with administered AS and AL exceeds the number of in-patients to whom these two drugs were dispensed. AS and AL are more in-demand than Q because; (i) AS is the drug of choice for its faster parasite clearance, has a less tedious administration regime, and safer profile and, (ii) AL is administered after both injectable AS and Q as the continuation of anti-malarial treatment in severe malaria [2].

Death could be attributed to severe malaria and/or to quinine-related treatment in the 88-year-old female with a single admission diagnosis of severe malaria. The caveat to this malaria-related attribution is diagnosis based on clinical judgement only (in the absence of microscopy results), unknown HIV-serostatus, advanced age, unknown random blood sugar levels and other comorbidities—especially cardiovascular comorbidities. That notwithstanding, Q was poorly administered at intervals of 23 h (between first and second doses) and 11 h (between second and third doses). Yet, 8-hourly intervals of injectable Q administration for at least 24 h are recommended until the patient is able to take oral medication [2]. The unconsciousness manifested in this in-patient is a known key sign of hypoglycaemia in severe falciparum malaria and carries a high risk of mortality [2]. Unfortunately, hypoglycaemia can result from both severe malaria and quinine-induced hyperinsulinaemia. Thus, blood sugar levels should be checked frequently in severe malaria in-patients who receive Q [2]. Also, this in-patient had tachycardia which could have resulted from Q use and/or hypoglycaemia. With hindsight, this elderly in-patient should have been treated with injectable AS instead, although the frequent unavailability of in-demand AS, and its associated higher cost, often dictates treatment with Q. This fatal case of suspected severe malaria underpins the need for the rapid turnaround of microscopy test-results. To further improve the clinical management of in-patients with severe malaria, the hospital should also invest in routine tests for random blood sugar, haemoglobin level/haematocrit, blood gases, urea and electrolytes; and in fluid balance charts as well as intensive nursing care.

The study limitations have been reported elsewhere [3]. Briefly, the study was conducted at Uganda’s National Referral and Teaching Hospital and the results might not be generalizable to facilities with lower calibres of in-patient care. Also, anti-malarials that were purchased from private community pharmacies were not documented as dispensed in the hospital register so we obtained this dispensing information by interviewing the in-patients and/or their caregivers [3].

## Conclusion

Half the malaria microscopy results were not available to guide the clinical management of malaria despite that the rate of testing was high. Seven in eight in-patients initiated on injectable AS or Q did not receive the recommended follow-up treatment of oral AL. One in four in-patients delayed to initiate hospital anti-malarials by at least one calendar day. The hospital should review its workflows to encourage prompt availability of malaria test-results to promote timely anti-malarial treatment based on confirmed diagnosis as opposed to clinical judgement only. Improved stock forecasting for in-demand anti-malarials, intern-pharmacist-led bed side dispensing and frequent audits of junior and mid-level clinicians could improve the quality of treatment for malaria in-patients in Uganda.

## Supplementary Information

**Additional file 1**: **Table S1**: Clinical details of 21 inpatients with a single admitting/discharge malaria diagnosis, Uganda, 2014. **Table S2**. Missed Day 1 dosing of quinine injection among 28 hospitalized patients who received in-hospital intravenous quinine, Uganda, 2014. **Table S3**. Patient-level risk factors for missed Day 1 dosing of administered antimalarials based on a 24-hour delay since admission among 72 inpatients with an admitting malaria diagnosis, Uganda, 2014.

## Data Availability

The dataset for this publication is available on reasonable request from the corresponding author.
