# Supplementary material for: Quality of care for adult in-patients with malaria in a tertiary hospital in Uganda
Source: Malar J. 2021 Apr 9;20:178. doi: 10.1186/s12936-021-03712-3 (PMC8034187; doi:10.1186/s12936-021-03712-3)
Supplement: Supplementary file 1 — Additional file 1: Table S1: Clinical details of 21 inpatients with a single admitting/discharge malaria diagnosis, Uganda, 2014. Table S2. Missed Day 1 dosing of quinine injection among 28 hospitalized patients who received in-hospital intravenous quinine, Uganda, 2014. Table S3. Patient-level risk factors for missed Day 1 dosing of administered antimalarials based on a 24-hour delay since admission among 72 inpatients with an admitting malaria diagnosis, Uganda, 2014. [file 12936_2021_3712_MOESM1_ESM.docx]

**Appendix**

Quality of Care for Adult Inpatients with Malaria in a Tertiary Hospital in Uganda

*Ronald Kiguba^1^*, Charles Karamagi^2^, and Sheila M. Bird^3^*

^1^Department of Pharmacology and Therapeutics, Makerere University College of Health Sciences, Kampala, Uganda

^2^Clinical Epidemiology Unit, Makerere University College of Health Sciences, Kampala, Uganda

^3^Medical Research Council Biostatistics Unit, Cambridge, United Kingdom

* Corresponding author

Email addresses:

RK: [kiguba@gmail.com](mailto:Kiguba@gmail.com)

CK: [ckaramagi2000@yahoo.com](mailto:ckaramagi2000@yahoo.com)

SMB: [sheila.bird@mrc-bsu.cam.ac.uk](mailto:sheila.bird@mrc-bsu.cam.ac.uk)

**Statistical analysis**

Time-to-first-dose of prescribed antimalarial treatment

Time-to-first dose of prescribed antimalarial treatment was measured by determining the time-interval between the date-and-time of hospitalization and the date-and-time of first dose of prescribed antimalarial after hospitalization. For inpatients in whom the first dose of antimalarial was administered within 24-hours preadmission, we computed the duration between date-and-time of first dose, if this information was available, and the date-and-time of hospitalization.

Parenteral-to-oral-switch of prescribed antimalarial treatment

We calculated proportions of inpatients who switched from injectable (artesunate, quinine) to oral (artemether-lumefantrine) antimalarials using, as numerator, the number of inpatients who were changed from parenteral to oral antimalarials and, as denominator, the total number of study patients who received parenteral antimalarials during the current hospitalization.

**Results**

Laboratory diagnosis of malaria

Microscopy for malaria parasitaemia was requested in 78% (65/83; 95% CI: 68% to 87%) of inpatients with both admitting and discharge malaria diagnoses; results were available for 51% (33/65; 95% CI: 38% to 63%) of the inpatients and tested positive in 61% (20/33; 95% CI: 42% to 77%) of them. Microscopy for malaria parasitaemia was requested in 72% (72/100; 95% CI: 62% to 81%) of the inpatients who received in-hospital antimalarials; results were available for 51% (37/72; 95% CI: 39% to 63%) of the inpatients, of whom 54% (20/37; 95% CI: 37% to 71%) tested positive. Only three of the 201 inpatients with microscopy requests had concurrent requests of malaria rapid diagnostic testing (RDT). However, the malaria RDT results were available for only one inpatient, who tested positive.

Frequently administered individual antimalarials

*Patient-level:* During the 4-weeks preadmission, oral artemether-lumefantrine (AL) only (52%, 50/97; 95% CI: 41% to 62%) was the most frequently administered antimalarial followed by intravenous or intramuscular quinine (Q) only (23%, 22/97; 95% CI: 15% to 32%), oral sulfadoxine-pyrimethamine (SP) only (9%, 9/97; 95% CI: 4% to 17%) and intravenous or intramuscular artesunate (AS) only (5%, 5/97; 95% CI: 2% to 12%), among others; see **Table 3**. In the current hospitalization, AS only (47%, 47/100; 95% CI: 37% to 57%) was the most frequently administered antimalarial followed by Q only (23%, 23/100; 95% CI: 15% to 32%), AL only (15%, 15/100; 95% CI: 9% to 24%) and AS + AL only (8%, 8/100; 95% CI: 4% to 15%), among others; see **Table 3**.

*Drug-level:* During the 4-weeks preadmission, AL (51%, 54/105) was the most frequently administered antimalarial followed by Q (27%, 23/105), SP (9%, 9/105) and AS (6%, 6/105), among others; see **Table 3**. In the current hospitalization, AS (50%, 57/113) was the most frequently administered antimalarial followed by Q (25%, 28/113) and AL (23%, 26/113); see **Table 3**.

Time-to-first dose of antimalarial treatment

Only 87 of the 100 inpatients with documented in-hospital antimalarial use had known date-and-time of administration of the first antimalarial dose, with 53 of the 87 inpatients having received AS.

One in seven (14%, 12/87; 95% CI: 7% to 23%) of the inpatients initiated antimalarials during the 24-hour period preceding the current hospitalization at a median pre-admission time of 9 hours (interquartile range, IQR, 4 to 17 hours) for all antimalarials, and 8 hours (IQR, 9 to 17 hours) for those who received AS (7/12). Of the 12 inpatients who initiated antimalarials during the 24-hours preadmission, six received AS only, three Q only, one AS + Q, one AL only, and one Q + AL.

The median time to post-admission antimalarial treatment initiation was 9 hours (IQR, 1 to 24 hours) for all antimalarials (75/87); and 7 hours (IQR, 10 to 40 hours) for inpatients who received AS (46/75). In 62 of the 75 inpatients who had an admitting malaria diagnosis, the median time to first dose for all antimalarials was 7 hours (IQR, 1 to 17 hours); and 5.5 hours (IQR, 1 to 20 hours) for 52 of the 62 who had both admitting and discharge malaria diagnoses.

Missed Day 1 dosing of hospital-prescribed antimalarials

*Calendar day:* A quarter (25%, 25/100; 95% CI: 17% to 35%) of the inpatients who received antimalarials during the current hospitalization missed their Day 1 dose of hospital-initiated antimalarials based on calendar day: being 24% (20/83; 95% CI: 15% to 35%) of the inpatients with an admitting diagnosis of malaria and 22% (16/72; 95% CI: 13% to 34%) of those with both admitting and discharge malaria diagnoses. Similar results of missed Day 1 dosing of antimalarials were observed based on *post-admission 24-hour delay* (see below).

*Post-admission 24-hour delay:* A quarter (25%, 19/75; 95% CI: 16% to 37%) of the inpatients who initiated antimalarials after being hospitalized, and in whom the time of first antimalarial dose administration was known, delayed to initiate antimalarials beyond 24 hours post-admission [median delay of 34 (IQR, 27 to 47) hours]: and was 23% (14/62; 95% CI: 13% to 35%) of inpatients with an admitting malaria diagnosis [median delay of 34 hours (IQR, 27 to 44 hours)] and 25% (13/52; 95% CI: 14% to 39%) of inpatients with both admitting and discharge malaria diagnoses [median delay of 36 hours (IQR, 27 to 44 hours)].

Missed Day 1 dosing classification by calendar day vs. *post-admission 24-hour delay*

The majority of inpatients (80%, 70/87; 95% CI: 71% to 88%) with documented time of first antimalarial dose administration had concordant classification of missed Day 1 dosing by both calendar day and *post-admission 24-hour delay* in antimalarial treatment initiation: 17% (12/70; 95% CI: 9% to 28%) missed Day 1 dosing and 83% (58/70; 95% CI: 72% to 91%) did not. Of the inpatients with discordant classification of missed Day 1 dosing, 59% (10/17; 95% CI: 33% to 82%) missed Day 1 dosing of antimalarials as measured by calendar day but not by *post-admission 24-hour delay* to antimalarial treatment initiation.

Parenteral-to-oral switch

The majority of inpatients to whom antimalarials were administered during hospitalization (83%, 83/100; 95% CI: 74% to 90%) received at least one form of parenteral antimalarial treatment during admission: AS (57/100); Q (28/100); both AS & Q (2/100). AL was co-prescribed for 65% (37/57; 95% CI: 51% to 77%) of the inpatients who received AS: 19% (7/37; 95% CI: 8% to 35%) switched from injectable AS to oral AL administration during the current hospitalization. AL was co-prescribed for 39% (11/28; 95% CI: 22% to 59%) of inpatients who received Q: 27% (3/11; 95% CI: 6% to 61%) switched from injectable Q to oral AL.

Medication use cycle

*Overview of the prescription, dispensing and administration of antimalarials*

*Artesunate*: AS was prescribed for 8% (62/762) of inpatients, dispensed to 85% (53/62), yet, administered in 57 inpatients (55 of 57 had an antimalarial prescription). AS was prescribed for 9 inpatients with malaria in pregnancy and dispensed and administered in all of them.

*Quinine*: Q was prescribed for 5% (40/762) of inpatients, dispensed to 70% (28/40) and administered in the 28 inpatients. Q was prescribed for 17 inpatients with malaria in pregnancy and dispensed and administered in 15 of them.

*Artemether-lumefantrine*: AL was prescribed for 9% (67/762) of inpatients [72% (48/67) of whom received in-hospital parenteral AS and/or Q)], dispensed to 33% (22/67), yet, administered in 28 inpatients (27 of 28 had an antimalarial prescription). More than two-thirds (69%, 46/67) of the inpatients received AL prescription at discharge [78% (36/46) of whom received AL co-prescription following in-hospital parenteral AS and/or Q)]; at least one dose of AL was administered in one-fifth (20%, 9/46) of the inpatients prior to hospital discharge [14% (5/36) of whom received in-hospital parenteral AS and/or Q)].

*Artemether*: AT was prescribed for two inpatients, dispensed to one and administered in none.

*Sulfadoxine-pyrimethamine*: SP was prescribed for five inpatients [four had Sickle Cell Disease (SCD)], dispensed to one (had SCD), yet, administered in two inpatients (both had SCD). None of the inpatients had malaria in pregnancy.

*Dihydroartemisinin-piperaquine*: DP was prescribed for two inpatients, yet, dispensed and administered in none.

*Incomplete dosing of in-hospital antimalarial treatment*

*Artesunate:* 19% (19/57; 95% CI: 10% to 32%) of the inpatients in whom *in-hospital AS was administered* had an *AS prescription* of <3 doses [median of 3 (IQR, 3 to 3)]; 47% (27/57; 95% CI: 34% to 61%) got <3 doses of *dispensed AS* [median of 2.5 (IQR, 1.5 to 3) doses]; 46% (26/57; 95% CI: 32% to 59%) received <3 doses of *administered AS* [median of 3 (IQR, 2 to 3) doses]; and 25% (14/57; 95% CI: 14% to 38%) received <3 doses of *both dispensed and administered AS*. For inpatients with malaria in pregnancy, 22% (2/9; 95% CI: 3% to 60%) received <3 doses of *both dispensed and administered AS*.

*Quinine:* None of the inpatients in whom *in-hospital Q was administered* had a *Q prescription* of <3 doses; 29% (8/28; 95% CI: 6% to 37%) got <3 doses of *dispensed Q* [median of 3 (IQR, 2 to 3) doses]; 46% (13/28; 95% CI: 28% to 66%) received <3 doses of *administered Q* [median of 3 (IQR, 2 to 3) doses]; and 21% (6/28; 95% CI: 8% to 41%) received <3 doses of *both dispensed and administered Q.* For inpatients with malaria in pregnancy, 13% (2/15; 95% CI: 2% to 40%) received <3 doses of *both dispensed and administered Q*.

*Artemether-Lumefantrine:* None of the inpatients in whom *in-hospital AL was administered* had an *AL prescription* of <6 doses and none got <6 doses of *dispensed AL*; 71% (20/28; 95% CI: 51% to 87%) received <6 doses of *administered AL* during hospitalization [median of 2 (IQR, 1 to 5) doses].

| **Table S1: Clinical details of 21 inpatients with a single admitting/discharge malaria diagnosis, Uganda, 2014** | | | | | | | | | | |
| --- | --- | --- | --- | --- | --- | --- | --- | --- | --- | --- |
| **Presenting complaints/cause of admission/Signs and symptoms** | **Admitting Diagnosis** | **Preadmission Antimalarial use** | **Microscopy for Parasitaemia** | **Microscopy Results** | **In-hospital Injectable Antimalarial use** | **Discharge Diagnosis** | **Length of Stay, days** | **ACTs Prescribed on Discharge** | **Discharge Outcome** | **Appropriate treatment*** |
| **Non-Severe Malaria Cases** | | | | | | | | | | |
| General body weakness, fever, chills, rigors and vomiting for 3 days | Malaria in pregnancy | - | Requested | Not Returned | Quinine (2 doses), Artesunate (3 doses) | Malaria in pregnancy | 4 | - | Discharged | No |
| Fever, chills, palpitations, backpain, frontal headache for 1 day | Malaria in pregnancy | Fansidar, 2 days prior to admission | Requested | Positive | Quinine (3 doses) | Malaria in pregnancy | 4 | AL | Discharged | Yes |
| Persistent fevers | Malaria | - | Requested | Negative | - | Septicaemia |  | - | Discharged | Yes |
| High grade fever | Malaria in pregnancy | - | Requested | Positive | Quinine (1 dose) | Malaria in pregnancy | 2 | AL | Discharged | No |
| High grade fever for 4 days | Clinical Malaria | - | Not requested | - | Quinine (2 doses) | Clinical Malaria | 2 | DP | Discharged | No |
| Palpitations & abdominal pain for 2-weeks; headache, vomiting & fever for 1-week | Malaria in pregnancy | - | Requested | Not Returned | Artesunate (2 doses) | Malaria in pregnancy | 1 | AL | Discharged | No |
| Fever for 3 days, headache and palpitations for 2 days, epistaxis | Malaria in pregnancy | - | Requested | Not Returned | Quinine (3 doses) | Malaria in pregnancy | 3 | AL | Discharged | Yes |
| Joint pain for 2 days; high grade fever, chills, rigors and headache for 1 day | Malaria in pregnancy | Quinine, 1 day prior to admission | Requested | Positive | Quinine (3 doses) | Malaria in pregnancy | 3 | - | Discharged | No |
| Headache and fever for 2-days | Malaria | - | Not requested | - | Artesunate (1 dose) | Malaria | 2 | AL | Discharged | No |
| High grade fever, vomiting, loss of appetite for 1-week | Poorly treated Malaria | AL, 1-week prior to admission | Requested | Not Returned | - | Malaria | 4 | - | Run away due to delayed investigations | No |
| Fever, vomiting and diarrhoea for 1-week | Malaria | Quinine, 5 days prior to admission | Not requested | - | Artesunate (3 doses) | Malaria | 4 | AL | Discharged | No |
| Fever & headache for 4-days | Malaria | - | Requested | Positive | Artesunate (3 doses) | Malaria | 3 | AL | Discharged | Yes |
| Fever and headache for 4-days | Malaria in pregnancy | - | Requested | Not Returned | Artesunate (2 doses), AL (1 dose) | Malaria in pregnancy | 2 | AL | Discharged | No |
| Wheezes and dyspnoea for 3-years; fever, headache, dizziness and joint pains for 1-week; vomiting for 1-day | Malaria in pregnancy | Quinine, on the day of admission | Requested | Positive | Artesunate (3 doses) | Malaria in pregnancy | 4 | AL | Discharged | Yes |
| Acute watery non-bloody diarrhoea (3 episodes), tinnitus and joint pain for 1-day | Malaria in pregnancy | Fansidar, 2-weeks prior to admission | Requested | Not Returned | Quinine (3 doses) | Malaria in pregnancy | 4 | - | Discharged | No |
| Vomiting, fever, chills, rigors, dizziness for 1-day | Malaria in pregnancy | Fansidar, 1-week prior to admission | Not requested | - | Quinine (3 doses), AL (1 dose) | Malaria in pregnancy | 5 | AL | Discharged | No |
| **Severe Malaria Cases** | | | | | | | | | | |
| Palpitations, dysuria, fever, headache for 3-weeks | Severe Malaria in pregnancy | - | Requested | Not Returned | Artesunate (2 doses) | Severe Malaria in pregnancy | 4 | - | Discharged | No |
| Fever, chills, loss of consciousness for 1 day | Severe Malaria | Quinine, 1-day prior to admission | Requested | Not Returned | Quinine (2 doses) | Severe Malaria | 2 | - | Death | No |
| Headache, Fever for 1-day | Severe Malaria | AL, 1-day prior to admission | Not Requested | - | Artesunate (2 doses) | Severe Malaria | 3 | AL | Discharged | No |
| High grade fever for 4-days, general body weakness for 2-days | Severe Malaria | AL, 4-days prior to admission | Not Requested | - | Artesunate (3 doses) | Severe Malaria | 7 | AL | Discharged | No |
| Fever, headache for 4-days | Severe Malaria | - | Requested | Not Returned | Quinine (2 doses) | Severe Malaria | 3 | AL | Discharged | No |
| AL is Artemether-Lumefantrine; DP is Dihydroartemisinin-Piperaquine; *Antimalarial treatment was considered inappropriate if the patient did not complete the 3-doses of parenteral AS or Q doses, a laboratory test for malaria parasitaemia was not requested, ACT was not co-prescribed following parenteral AS or Q or if antimalarials were administered without a malaria diagnosis; Only 5 of 21 inpatients received appropriate antimalarial treatment; none of the 5 severe malaria inpatients received appropriate antimalarial treatment. | | | | | | | | | | |

| **Table S2: Missed Day 1 dosing of quinine injection among 28 hospitalized patients who received in-hospital intravenous quinine, Uganda, 2014** | | | | | | | | | | | |
| --- | --- | --- | --- | --- | --- | --- | --- | --- | --- | --- | --- |
| **Length of stay, days** | **Preadmission antimalarial use** | **Admission Malaria Diagnosis** | **Discharge Malaria Diagnosis** | **AL co-prescribed** | **Switched to AL** | **Doses Prescribed** | **Doses Received** | **Day 1** | **Day 2** | **Day 3** | **Day 4** |
| 2 | No | Yes | Yes | Yes | No | 3 | 2 | 1 dose | 1 dose |  |  |
| 6 | Yes | Yes | Yes | Yes | No | 3 | 3 | 1 dose |  | 1 dose | 1 dose |
| 8 | Yes | No | No | No | No | 3 | 1 | 1 dose |  |  |  |
| 4 | Yes | Yes | Yes | No | No | 3 | 3 | 1 dose | 1 dose | 1 dose |  |
| 3 | No | Yes | Yes | No | No | 3 | 3 | 1 dose | 2 doses |  |  |
| 4 | No | Yes | Yes | No | No | 3 | 1 | 1 dose |  |  |  |
| 3 | No | Yes | Yes | No | No | 3 | 2 | 1 dose | 1 dose |  |  |
| 2 | Yes | Yes | Yes | No | No | 3 | 2 | 1 dose | 1 dose |  |  |
| 2 | Yes | Yes | Yes | Yes | No | 3 | 1 | 1 dose |  |  |  |
| 3 | No | Yes | Yes | Yes | No | 3 | 2 | 1 dose | 1 dose |  |  |
| 5 | Yes | Yes | Yes | No | No | 3 | 3 | 2 doses | 1 dose |  |  |
| 3 | Yes | Yes | Yes | No | No | 9 | 3 | 1 dose | 1 dose | 1 dose |  |
| 4 | No | Yes | Yes | No | No | 3 | 3 | 1 dose | 1 dose | 1 dose |  |
| 3 | No | Yes | Yes | Yes | Yes | 3 | 3 | 1 dose | 2 doses |  |  |
| 4 | No | Yes | Yes | No | No | 6 | 2 | 1 dose | 1 dose |  |  |
| 8 | No | Yes | Yes | No | No | 3 | 3 | 1 dose | 2 doses |  |  |
| 5 | Yes | Yes | Yes | Yes | Yes | 3 | 3 | 1 dose |  | 1 dose | 1 dose |
| 4 | Yes | Yes | Yes | Yes | No | 3 | 3 | 3 doses |  |  |  |
| 3 | Yes | Yes | Yes | No | No | 9 | 3 | 2 doses | 1 dose |  |  |
| 2 | No | Yes | Yes | No | No | 3 | 2 | 1 dose | 1 dose |  |  |
| 9 | No | Yes | No | No | No | 6 | 3 |  | 2 doses | 1 dose |  |
| 3 | No | Yes | Yes | Yes | No | 21 | 3 | 2 doses | 1 dose |  |  |
| 2 | No | Yes | Yes | Yes | No | 0 | 1 | 1 dose |  |  |  |
| 10 | No | Yes | Yes | Yes | Yes | 6 | 3 | 2 doses | 1 dose |  |  |
| 15 | Yes | No | Yes | No | No | 21 | 3 |  |  | 2 doses | 1 dose |
| 2 | No | Yes | Yes | No | No | 3 | 2 | 1 dose | 1 dose |  |  |
| 2 | No | Yes | Yes | No | No | 3 | 2 | 1 dose | 1 dose |  |  |
| 6 | No | Yes | Yes | Yes | Yes | 3 | 2 | 1 dose | 1 dose |  |  |
| **Missed-dose day, n** | | | | | | | | 5 | 2 | 1 |  |
| **Dose-day data unavailable, n1** | | | | | | | | 0 | 8 | 21 |  |
| **Dose-day data available, N *** | | | | | | | | 28 | 20 | 7 |  |
| **Proportion of inpatients with missed Day 1, n/N ~** | | | | | | | | 18 |  |  |  |
| *N = [(The 28 quinine users) – (Number of inpatients without quinine dose-day data)] or (28-n1); ~95% confidence intervals for the estimate is 18% (6% to 37%). | | | | | | | | | | | |

| **Table S3: Patient-level risk factors for missed Day 1 dosing of administered antimalarials based on a 24-hour delay since admission among 72 inpatients with an admitting malaria diagnosis, Uganda, 2014** | | | | | | | | | | |
| --- | --- | --- | --- | --- | --- | --- | --- | --- | --- | --- |
| **Missed Day 1 dosing of antimalarials by 24-hour Day, n (%); (N = 72) *** | | | | | | | | | | |
| **Factor** | **Missed 24-hour Day 1 dosing** | | | | **Crude Analysis** | | | **Adjusted Analysis** | | |
|  | **Yes** | **No** | **Total, [% col]^a^** | **Crude** | | | **Adjusted** | | | |
|  |  |  |  | OR^b^ | 95% for CI | *P*-value | OR^b^ | | 95% for CI | *P*-value |
| Antiretroviral therapy use | | |  |  |  |  |  | |  |  |
| No | 11 ( 6) | 52 (94) | 63 [88] | 1.0 |  |  | 1.0 | |  |  |
| Yes | 3 (24) | 6 (76) | 9 [12] | 2.4 | 0.51-10.9 | 0.271 | 1.9 | | 0.34-10.7 | 0.470 |
|  |  |  |  |  |  |  |  | |  |  |
| Laboratory test results for malaria | | |  |  |  |  |  | |  |  |
| Not available | 5 (11) | 40 (89) | 45 [63] | 1.0 |  |  | 1.0 | |  |  |
| Available | 9 (33) | 18 (67) | 27 [37] | 4.0 | 1.17-13.6 | 0.027 | 3.6 | | 0.96-13.3 | 0.058 |
|  | | |  |  |  |  |  | |  |  |
| Linear on number of working | | |  |  |  |  |  | |  |  |
| Diagnoses | 14 | 58 | 72 | 1.8 | 1.18-2.71 | 0.006 | 1.8 | | 1.11-2.76 | 0.016 |
| ^a^% Column; ^b^OR = Odds Ratio; *24-hour delay data were available for only 72 of the 83 inpatients with an admitting malaria diagnosis and who received in-hospital antimalarial treatment. | | | | | | | | | | |
